# Evaluating Guayule (*Parthenium argentatum* A. Gray) Germplasm Grown in Spain: Rubber and Resin along Three Production Cycles

**DOI:** 10.3390/plants13081092

**Published:** 2024-04-13

**Authors:** Francisco Miguel Jara, María de las Mercedes García-Martínez, Horacio López-Córcoles, María Engracia Carrión, Amaya Zalacain, Manuel Carmona

**Affiliations:** 1 Instituto Técnico Agronómico Provincial (ITAP), S.A. Polígono Industrial Campollano, Avenida 2, 42 B, 02007 Albacete, Spain; fjg.itap@dipualba.es (F.M.J.); mariamercedes.garcia@uclm.es (M.d.l.M.G.-M.); hlc.itap@dipualba.es (H.L.-C.); 2 Cátedra de Química Agrícola, Escuela Técnica Superior de Ingeniería Agronómica, de Montes y Biotecnología (ETSIAMB), Universidad de Castilla-La Mancha, Avda. de España s/n, 02071 Albacete, Spain; amaya.zalacain@uclm.es; 3 Food Quality Research Group, Institute for Regional Development (IDR), Universidad de Castilla-La Mancha, Campus Universitario s/n, 02071 Albacete, Spain; mengracia.carrion@uclm.es

**Keywords:** guayule, germplasm, rubber, resin, production, *Parthenium argentatum* A. Gray, Spain

## Abstract

Rubber and resin are potentially important products of guayule (*Parthenium argentatum* A. Gray) that can assure the profitability of this crop as an agricultural alternative for the semi-arid areas of central and eastern Spain. This study analyzes, for the first time, the changes in rubber and resin production across 27 guayule accessions (traditional and modern) and along three cycles under the agroclimatic conditions of Castilla-La Mancha, simulating industrial management with a biannual harvest. The rubber content (% of dry mass) increased from 4.2% in one-year-old plants to 6.6% in two-year-old plants, but decreased after harvesting. Contrastingly, the rubber yield doubled in contiguous sampling, reaching a mean of 303.6 kg ha^−1^, with a maximum yield of 341.2 kg ha^−1^ after the first harvest. Three patterns of rubber production were established based on the production periods. A similar analysis was performed for resin production, which was heterogeneous across accessions belonging to the same rubber groups. In this case, three independent groups were established to classify the resin accumulation profiles following the same criteria of production periods. Here, we demonstrate that biannual harvesting has the potential to enhance rubber accumulation in summer, although more research is needed for its adaption to current harvesting techniques in this area.

## 1. Introduction

Guayule (*Parthenium argentatum* A. Gray) is a promising alternative crop for semi-arid areas with limited irrigation [1,2] and has the potential to replace, at least in part, the supply of natural rubber, which is currently produced exclusively from the rubber tree *Hevea brasiliensis* [3]. Natural rubber is used to manufacture many thousands of essential products, including molded goods such as tires and hoses, and medical products such as gloves, catheters and feeding nipples [4]. The superiority of natural rubber over synthetic forms (produced from petroleum) comes from its resilience, elasticity, abrasion resistance, efficient heat distribution, impact resistance and malleability at cold temperatures [5]. Although the industrial commercialization of guayule is presently limited, strategies for the valorization of rubber, resin and other co-products are being developed in the setting of retired land with adverse environmental conditions and irrigation [2]. Indeed, in addition to rubber, the economic viability of guayule will depend on the successful exploitation of its many co-products [6,7,8]. Guayule resin, which is produced by the plant in considerably greater amounts than rubber in almost all of the guayule varieties developed in the 1980s and 1990s [9,10], is one of the more marketable co-products, as it contains a wide spectrum of secondary metabolites (sesquiterpene esters, triterpene alcohols and fatty acids) with a myriad of industrial applications [6,11]. About 50 secondary metabolites have been identified thus far [12], and they are known to vary in abundance depending on the specific plant accession, and also depending on the harvesting date, cultivation site and processing history [6,13].

Rubber production in guayule begins after the second year and the breakeven point is the fourth year [7]. It is well established that the single most important factor for optimal rubber accumulation in guayule is the drop in the nighttime temperature that occurs in winter [14,15,16]. Other important factors include the sudden restriction of available nitrogen [14], the length of the day [17], the light intensity [16] and the water availability [18]. Although there is general agreement on the induction of rubber production by cold temperatures, there is debate about the average night temperatures that stimulate rubber production. Early studies reported an activation temperature of 7–10 °C [14,15,17,19], and later studies determined that a drop to 15 °C [16] or 20 °C [20] was necessary. These differences are likely because many of the studies were performed with young plants, with only a few using adult plants—for example, plants at 18–36 months [17] or 36 months [16] of age. It is now known that plants must be at least 200 days old for temperature induction to be activated [20]. In contrast to rubber, much less is understood about the factors involved in resin production. Indeed, the push of the industry and the memory of natural rubber shortages in times of crises [8] has led to the creation of vast knowledge about guayule rubber accumulation, but the patterns of resin accumulation have received scant attention. 

Recent advances in extraction processes for rubber, resins and other co-products have fueled renewed efforts for commercial guayule cultivation in potential growing regions, such as the semi-arid regions of Castilla-La Mancha (Spain). These new locations, however, need to be tested not only because of the different environmental requirements of each accession [21], but also because more productive accessions have been developed in terms of both natural rubber and resin, particularly by researchers of the Agricultural Research Service (ARS) of the United States Department of Agriculture (USDA) and the University of Arizona [22,23]. Previous references of traditional guayule accessions cultivated in Spain in the 1950s and 1960s, should be compared in the same cultivation area with the accessions developed later. Recently, the adaptation of 20 pure guayule accessions, traditional (9) and modern ones (11), together with seven hybrids, to the agroclimatic conditions of Castilla-La Mancha was investigated by comparing their vegetative characteristics across three sampling cycles [2], confirming that genetic imprinting remained important until plants were two years old; after this, time environmental effects were dominant in the second and, particularly, the third cycle during biannual harvesting. Also, the overall biomass production profile along the three cycles was unique to the germplasm tested, the harvest management practices and the agroclimatic conditions. However, in addition to biomass, the production of potentially useful co-products should be considered when selecting the best germplasm for a new growing area [2]. In this respect, the present study analyzed, for the first time, the accumulation profile of rubber and resin in the same variety of guayule germplasms (20: 9 pure traditional accessions and 11 modern pure ones), together with some modern hybrids (seven) that were adapted for growth in Santa Cruz de la Zarza (Toledo, Spain), following a schedule of 24, 48 and 60 months after planting. The purpose of the study was to compare the accumulation profiles of the rubber and resin provided by these accessions under the severe climatic conditions of this area across the three cycles before and after biannual harvesting. This information will be helpful in selecting those lines that best adapt to the agroclimatic conditions of Castilla-La Mancha for higher rubber and resin production when the crop is used for industrial production.

## 2. Results

Both the content of rubber (Rb) and resin (Rs) in plants and the yield of their production per hectare in branches (YRb and YRs) were studied along three cycles, simulating biannual harvesting for industrial management. The first analysis was performed from 13 to 24 months after transplanting, a period when a large portion of the observed variation is attributed to genetic effects [9,24]. The second analysis was performed from the plant harvest at the 24th month (end of cycle 1) to the new harvest two years later (cycle 2; 25 to 48 months), a period when competition between plants and environmental effects mask the genetic imprint of each accession [9,24]. The third period of study was from the plant harvest at the 49th month (end of cycle 2) to the new outgrowth almost one year later, from 49 to 60 months.

### 2.1. Rubber Production

The rubber content was studied across six samplings and three crop cycles to investigate its variability in the 27 accessions cultivated in the same plot (Table 1). 

The mean rubber content (Rb) increased from 4.2% in S1 (month 13) to 6.6% in S3 (month 24) in the first cycle. This period represented the greatest production period of rubber in the plants, as it decreased to a mean of 5.7% at the end of the second cycle (S4R, month 48), 24 months after regrowth, and to 3.8% one year after the second regrowth (S6R, month 60). These findings suggest that the rubber content (percentage) in plants decreases after regrowth. Contrastingly, the yield of rubber production (YRb) doubled in contiguous sampling along the first cycle (S1: 60.6 kg ha^−1^, S2: 134.6 kg ha^−1^ and S3: 303.6 kg ha^−1^). The differences between the minimum and maximum yields of the different accessions were very large and increased over time. The maximum yield was observed after the first regrowth in S4R (341.2 kg ha^−1^), which was comparable with plants cultivated for the same time (24 months) before harvesting (S3). The same increment was true for plants grown for 12 months after the second harvest (S6R, 174.4 kg ha^−1^) compared with 12-month-old plants (S1, 60.1 kg ha^−1^). This is reasonable to expect, as the plant has larger roots after each growth period and can resprout more vigorously. However, when considering all accessions together, there was an unexpected decrease in the mean YRb between S5R and S6R that is likely linked to the heterogeneous performance of the different accessions.

Rubber production was dependent on the age of the plant and the period of the cycle, but was mainly dependent on the accession. As shown in Table 2, the accessions that produced the most rubber, with a content over 10% at the period of maximum rate (S3), were traditional accessions such as 11635 (11.4%), 11590 (10.3%), A48118 (10.3%) and N565 (10.1%). The same accessions also exhibited the maximum yields at this sampling period, with 566.4 kg ha^−1^, 428.8 kg ha^−1^, 602.1 kg ha^−1^ and 400.1 kg ha^−1^, respectively. However, it should be noted that a higher content (in percentage terms) does not always equate to more yield per hectare, and vice versa, as the generated biomass is also an important parameter in the yield equation. For instance, the yield for the modern accession genotype AZ-5 was 517.4 kg ha^−1^ with 9.0% rubber content, outperforming 11590 and N565 even though they had a higher percentage (10.3–10.1%, respectively). A rubber content of 9% was also achieved in previous studies of this crop carried out in the 1960s in Spain at a more southerly location with some traditional guayule genotypes [25]. Overall, the yields obtained for the 27 accessions, pure or hybrids, did not reach the cut-off value to be considered profitable enough for a potential commercial facility in Europe to produce latex, crude rubber, resin and bagasse as final products, which has been established at 810 kg ha^−1^ year^−1^ of natural rubber (90 tons of total dry biomass in 10-year cultivation cycle containing 9% of natural rubber) [7]. Moreover, the expected production yield of the crop would not reach the level obtained in its original geographical location, which has been estimated at 1400 kg ha^−1^ year^−1^ in the USA [26]. As mentioned in our previous study about biomass production [2], the reason could be that the irrigation supplied was significantly lower compared with other studies. The amount of irrigation that the plantation received over 4 years (2304 mm) was less than half of what some authors consider deficit irrigation (2519 mm in 2 years) [27] and far from what is considered the optimum irrigation to achieve the maximum productive rate (3357–3573 mm in 2 years) [28,29].

Indeed, most germplasms used in the present study underperformed when considering Rb and YRb, and only the five aforementioned accessions (11635, 11590 [CL1], A48118, N565 and AZ-5) achieved a rubber content of 9% in S3; many reached a maximum of only 3.0–3.3% rubber, which is considerably inferior to that considered as profitable. 

Other authors have also reported rubber production yields that are below these expectations. For example, Coffelt et al. [3] studied rubber production in AZ-1, AZ-3 and AZ-5, observing a mean percentage of rubber of 4.2, 3.8 and 5.5%, respectively, when transplanted in spring. Likewise, Foster et al. [22] reported low percentages of rubber production for AZ-1 and AZ-3 in three-year-old plants (2.6 and 2.5%, respectively), although the yields of 824 and 717 kg ha^−1^ were acceptable based on the theoretical expectations (810 kg ha^−1^ year^−1^). Likewise, Wang et al. [30] reported percentages between 2 and 5% in AZ-2 at two years depending on the irrigation regime, which is similar to the results of this study for plants in the same age range before and after harvesting (4.0% in S3 and 5.2% in S4R). 

With regard to the traditional accessions, a yield of 552 kg ha^−1^ of rubber was reported for N565 in Australia at 15 months [10], which compares well with the maximum production yields of 400.1 kg ha^−1^ in S3 and 421.7 kg ha^−1^ in S4R, 24 months before and after harvesting. These differences are likely due to the agroclimatic conditions, as the influence of the environment could be critical even if the accessions are adapted to new growing regions. In this scenario, managing guayule to increase rubber production as well as valorizing other final products becomes relevant.

### 2.2. Resin Production

The content of resin was also studied along the three crop cycles in the same accessions and experimental plot. The mean resin content (Rs) was higher than that for rubber at all sampling points and was occasionally double or triple the yield (Table 1). The highest Rs was found in S2 (12.5%) in 12-month-old plants before harvesting. A comparative analysis of 24-month-old plants before and after the first harvest (S3 versus S4R) revealed that the resin content after harvesting was slightly higher (11.8% in S4R versus 10.0% in S3). This would suggest that harvesting favors the accumulation of resin, perhaps as a defense response to the abiotic stress caused. However, the yield of resin production increased continuously and significantly from S1 to S3 along the first cycle (S1: 167.5 kg ha^−1^, S2: 421.8 kg ha^−1^ and S3: 479.1 kg ha^−1^). Similar to the results for rubber, the maximum yield for resin was found after the first harvest in S4R at 720.12 kg ha^−1^, which was higher than for plants at the same age before harvesting (S3: 479.12 kg ha^−1^). After the second harvest, the mean yield was lower than for S4R, and was similar to that for plants at the same age before the first harvest (348.9 kg ha^−1^ in S6R versus 421.8 kg ha^−1^ in S2). 

Resin production was also dependent on the accession (Table 3) and was greatest for the modern pure accessions AZ-5 (19.2%), R1108 (18.1%), AZ-1 (16.7%) and the traditional pure accession 11604 (16.0%) in S2, which is the period of maximum production. However, the maximum yields were observed at S4R, 24 months after the first harvest, with values of 912.5 kg ha^−1^, 416.1 kg ha^−1^, 654.5 kg ha^−1^ and 591.2 kg ha^−1^, respectively. Similar to the results for rubber content, the greater percentage of resin content did not always equate to more yield per hectare and vice versa, as exemplified by the modern hybrid CAL-1, whose yield was 553.6 kg ha^−1^ with 7.5% in S3.

The improvement of traditional accessions in order to increase their resin content resulted in accessions such as AZ-1, AZ-2, AZ-3, AZ-5 and AZ-6, whose superiority was mostly evident when compared with ancient accessions such as 593 or N565. As previously reported by others [9,10], the AZ-type accessions were larger in size and produced more resin than N565. In the case of 593, the YRs was lower than that for all AZ-accessions at all sampling points. That being said, the resin production at S3, S4R and S5R was greater in the traditional pure accession N565 than in modern pure accessions such as AZ-6, and was greater than in the hybrids AZ-1 and AZ-2 in S3. The germplasm with the greatest YRs was AZ-2, with 1174.7 kg ha^−1^ in S4R. All AZ lines and AZ-2 in particular were better resin producers than the traditional accessions based on the results of Coffelt [31]. 

While the resin content in the studied accession was higher than that reported by other authors, the yield was lower. For instance, Foster et al. [22] reported lower values of resin content for AZ-1, AZ-2 and AZ-3 (around 6% for two-year-old plants and 7.5% for three-year-old plants), although the yields (655–778 kg ha^−1^ for two-year-old plants and 2097–2388 kg ha^−1^ for three-year-old plants) were much higher than those obtained in the present study (Table 3). Beyond the accession *per se*, the age of the plant, the period of the cycle, and the temperature could be key for the accumulation of natural rubber in guayule. As this is not known for resin, the profiles of accumulation for both rubber and resin in relation to temperature were studied in this industrial management scenario based on biannual harvesting.

### 2.3. Patterns of Rubber Production

**Figure 1:**
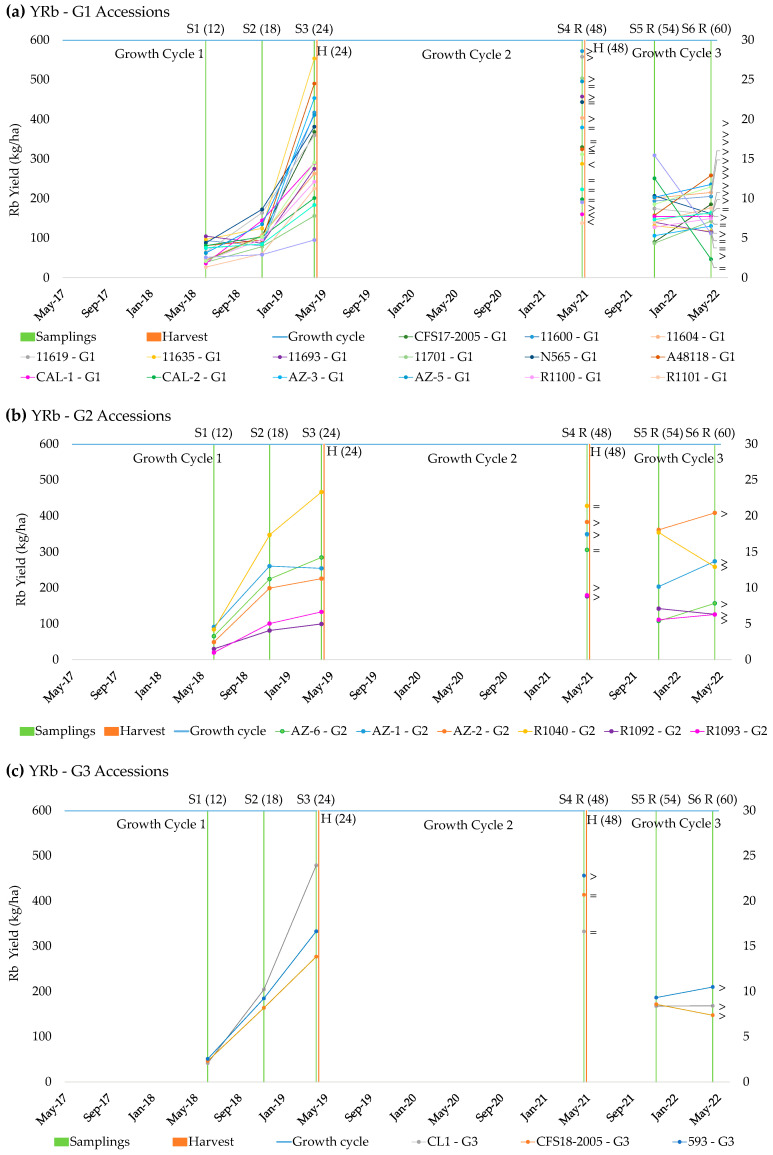
Accumulation of rubber yield (YRb) in sampling times (S1–S6R) by accessions in three groups (**a**–**c**). S1 (12), May 2018 (12-month-old plants); S2 (18), November 2018 (18-month-old plants); S3 (24), April 2019 (24-month-old plants); S4R (48), April 2021 (48 months after transplanting, 24 months after harvesting); S5R (54), December 2021 (54 months after transplanting, 6 months after harvesting); S6R (60), April 2022 (60 months after transplanting, 12 months after harvesting).

Knowledge of the pattern of accumulation of rubber and resin along the three cycles should provide useful information to optimize production and determine the best harvesting time. As shown in Figure 1 and Table 4, the production of rubber was dependent on the accession. Three main groups of accessions could be identified based on significant differences (ANOVA) in the accumulation pattern in the first cycle, when plants were 24 months old. 

The first group (G1, Figure 1) comprised 12 guayule pure accessions, the nine traditional ones (11600, 11604, 11619, 11635, 11693, 11701, N565 and A48118) and the other four modern ones (CFS17-2005, AZ-5, R1108, and CAL-7), together with six modern hybrids (CAL-1, CAL-2, AZ-3, R1100, R1101 and R1103), whose common characteristics were that the rubber production tended to increase from S1 (June 2018) to S2 (November 2018), and that it significantly increased in S3 (April 2019) (Table 3). Within this group, five accessions displayed a slightly different behavior: 11604, 11619 and CAL-2 showed the same level of production between S2 and S3, and the levels of rubber in 11701 and R1103 were not significantly different between the three samplings S1–S3. Examination of the evolution of rubber production in the second cycle after the first harvest revealed that the level of rubber production was the same for 24-month-old plants before and after harvesting (S3 versus S4R) for eight accessions (denoted as = in Figure 1). Contrastingly, four accessions (11635, A48118, CAL-1 and R1101) showed a decrease in the rubber content in S4R, while six accessions (11600, 11604, 11619, 11693, 11701 and R1103) showed an increase (Figure 1).

After the second harvest, in the third cycle, 12-month-old plants produced more rubber after harvesting (S6R) than before (S1) on average. As a general observation, accessions in this group accumulated rubber in winter, regrew well after harvesting and reached the same levels of rubber production at 24 months but with a greater production at 12 months. This suggests that harvesting would favor the accumulation of rubber in summer. 

The second group (G2, Figure 1) comprised five guayule modern pure accessions (AZ-1, AZ-6, R1092, R1093 and R1040) and one hybrid (AZ-2) that showed a significant increase in rubber production in S2, after the summer (Table 4). However, they failed to accumulate rubber in winter, as the production in S3 was the same as in S2. R1092 and AZ-2 showed the same trend, but the rubber production was not significantly different across samplings S1–S3. Contrastingly, rubber accumulation in the hybrid accession R1040 was only significantly different between S1 and S3. Regarding the evolution of rubber production in the second cycle (after the first harvesting), the results showed an increase for 24-month-old plants after harvesting (S4R versus S3) in most accessions (AZ-1, AZ-2, R1092 and R1093). The positive impact of harvesting on rubber production was more evident in this group, especially for AZ-2, which was found to be very productive in other studies [23,29,30], but which did not respond well to the soil and climatic conditions during the first 24 months. Of note, harvesting the entire aerial part of the plant when it is two years old can increase the yield of rubber and might stimulate faster regrowth following harvesting [28,30]. After the second harvest, in the third cycle, 12-month-old plants produced more rubber after harvesting (S6R) than before (S1). Overall, it was evident that accessions in this group accumulate rubber in summer and regrow well after harvesting to achieve the same levels of production as 24-month-old plants, but with a higher production at 12 months since the accumulation of rubber in summer is improved. These results are somewhat surprising because it was perceived wisdom that rubber production required activation by low temperatures during the winter [17,19], with the caveat being that this minimum temperature has increased with successive studies to 20 °C, which was established in the most recent works [16,20].

Three guayule accessions comprised the third group (G3, Figure 1): 11591 (CL1), CFS18-2005 and 593; these were neither traditional nor modern accessions. The pattern of rubber accumulation in G3 was characteristic because of the progressive increase in rubber from S1 to S3, with significant differences between the three samplings (Table 4); this is with the exception of the pure modern accession CFS18-2005 between S1 and S2. In the second cycle, after the first harvest, the level of rubber production in 11591 (CL1) and CFS18-2005 was the same for 24-month-old plants before and after harvesting (S3 versus S4R), whereas the traditional 593 showed an increased rubber content in S4R. After the second harvest, in the third cycle, 12-month-old plants produced more rubber after harvesting (S6R) than before (S1). These results indicate that accessions in this group accumulate rubber in both summer and winter and regrow well after harvesting to achieve the same levels of production in 24-month-old plants, but with a higher production at 12 months. Again, it seems that the accumulation of rubber in summer is improved by harvesting.

**Figure 2:**
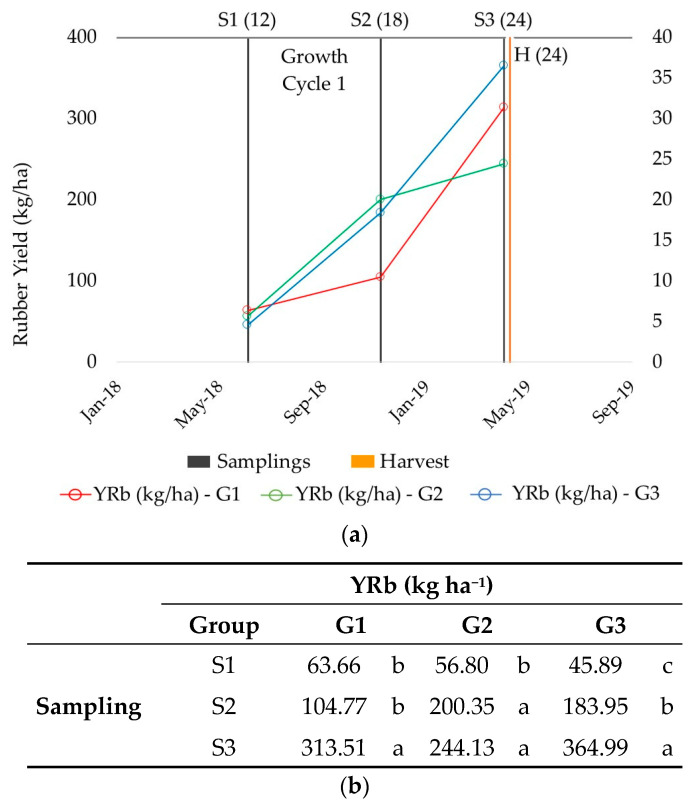
Patterns of accumulation of rubber yield (YRb) according to the groups (G1, G2 and G3) established related to the sampling times (S1–S3) within the first growth cycle, prior to harvesting. (**a**) Graphical representation and (**b**) statistical significance between samplings. S1 (12), May 2018 (12-month-old plants); S2 (18), November 2018 (18-month-old plants); S3 (24), April 2019 (24-month-old plants). Different letters indicate significant differences among samplings. Tukey test, *p* value <0.05.

A summary of the patterns of accumulation and average yields of the three groups is depicted in Figure 2, revealing that accessions in G1 accumulate rubber in winter. Although it would seem that accessions in G1 are poorer rubber producers than those in G2 and G3, they actually start producing rubber later. In fact, the levels of production in this group could reach and even improve upon the other groups after 24 months. All interspecific hybrids except AZ-2 (G2) are classified in this group (G1). In the work of Cornish and Backhaus [20], which was performed to establish the temperature at which rubber starts to be produced, they used two of the traditional varieties (11591 and 593) classified in our study as G3 (producing both in summer and winter), which led them to suggest that the temperature was higher than that determined so far. Sundar et al. [16] also used 11591 to reach the same conclusion a few years earlier; this is that the temperature at which rubber can be produced is higher than previously thought, so rubber can be generated all year round. On the contrary, in G1, there are traditional varieties together with hybrids that would not behave in this way, only producing rubber during the winter; this is why the selection of varieties must take into account the environmental temperature cycles over a long period of time (10 years).

For example, the accessions in G2 accumulate rubber in summer, at higher levels than the accessions in G1 in S2. However, the rubber production is reduced in winter, a period when G1 is likely producing rubber due to being activated by cold temperatures, as the plants are older than 200 days [20]. Accordingly, G2 starts producing rubber early, but production slows after the Summer. Finally, G3 accumulates rubber both in summer and winter. Although this group might appear to be the highest rubber producer (on average), the groups are not associated with the level of yield but rather with the evolution in the growth cycle. In fact, accessions such as 11604 or R1040, belonging to G1 and G2, respectively, produce more rubber than CFS18-2005 (G3) in S3, with 310.77 kg ha^−1^ and 484.22 kg ha^−1^, respectively, versus 265.50 kg ha^−1^ (Table 2).

### 2.4. Patterns of Resin Production

**Figure 3:**
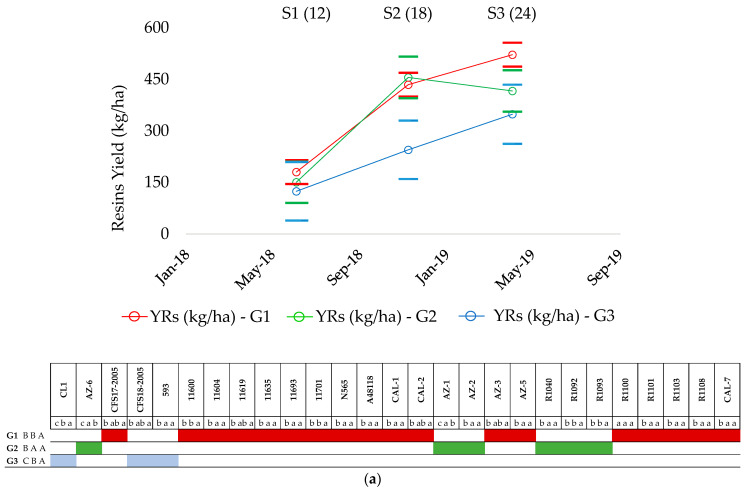

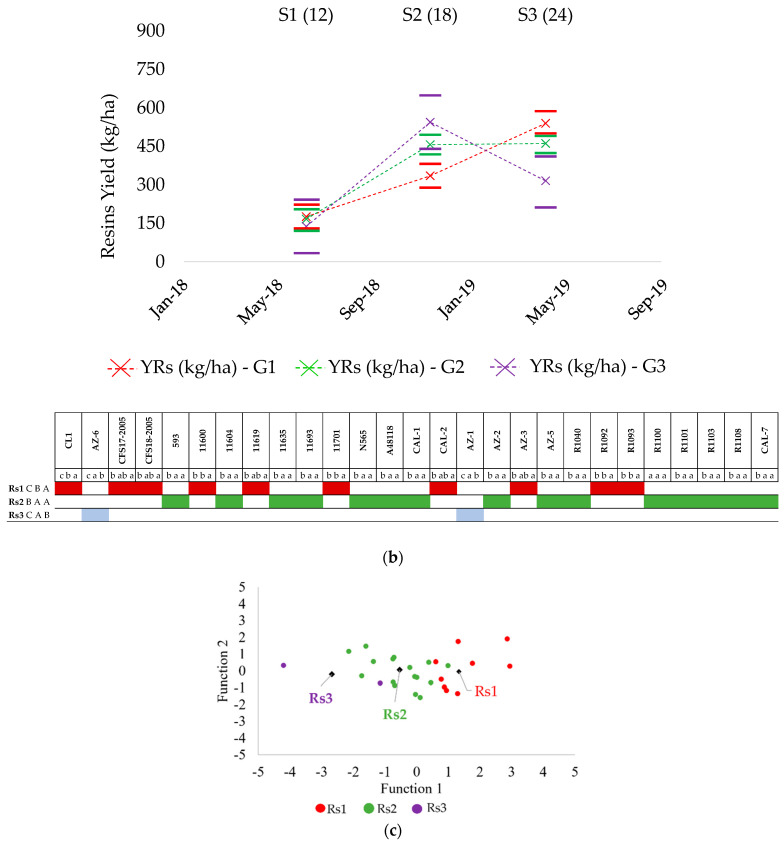
(**a**) Patterns of accumulation of resin yield (YRs) by group (G1 to G3), established based on rubber accumulation within the first growth cycle, prior to harvesting. (**b**) New patterns of accumulation of YRs proposed and related to resin evolution (Rs1, Rs2 and Rs3) within the first growth cycle (S1–S3), prior to harvesting; (**c**) Canonic discriminant functions to validate new resin groups, with 85.2% of original cases correctly grouped. S1 (12), May 2018 (12-month-old plants); S2 (18), November 2018 (18-month-old plants); S3 (24), April 2019 (24-month-old plants). Capital letters represent patterns of evolution of resin production within every group (GX or RsX). Small letters represent patterns of evolution of resin production by accession. Different letters signify significant differences within sampling in the first growth cycle (S1, S2 and S3) with regard to ANOVA performed by Tukey test, *p* < 0.05. Short solid lines means the max and min error for every sampling point; “o” and “x” markers refers to the mean value measured.

The three groups established based on rubber production displayed different profiles of resin accumulation (Figure 3a) among accessions and did not take into account their origin (pure or hybrids, traditional or modern). In the case of G1 and G3, the resin content increased progressively from S1 to S3, with significant differences between the three sampling times. Contrastingly, G2 showed a significant increase in the resin content from S1 to S2, which was maintained in S3. Analysis of the individual profiles of the accessions across S1–S3 revealed that the accessions grouped together based on their rubber profile behaved differently when based on their resin profile (Figure 3a). For example, using the group with the fewest members as a reference (G3, 3 members), the results clearly show that all accessions behaved differently. While resin production in 11591 (CL-1) increased throughout the sampling (S1, c; S2, b; S3, a), in the case of 593, it increased from S1 to S2 and then leveled off; in the case of CFS18-2005, differences were only observed between S1 and S3. Accordingly, a new classification based on the resin profile was developed (Figure 3b). 

As before, three different groups (Rs1, Rs2 and Rs3) that correctly classified 85.2% of the accessions were obtained (Figure 3c). The first group (Rs1) comprised eight pure guayule accessions, two traditional (11600, 11619) and six modern accessions (11591 (CL1), CFS17-2005, CFS18-2005, R1092, and R1093) and two hybrids (AZ-3 and CAL-2). This group was characterized by a progressive increase in the resin yield from S1 (12 months after transplanting) to S3 (CBA profile attending to statistical significance). 

The second group (Rs2) was characterized by a significant increase in the resin yield in S2 (18 months after transplanting) and by the maintenance of this level in S3, when the plants were 24 months old (BAA profile attending to statistical significance). Ten pure, six traditional (593, 11604, 11635, 11693, N565, A48118), four modern (AZ-5, R1040, R1108 and CAL-7) and five hybrid accessions (CAL-1, AZ-2, R1100, R1101 and R1103) belonged to this group. Finally, the modern pure accessions AZ-1 and AZ-6 were classified in a third group (Rs3), as their resin yield increased in S2 and then decreased in S3, with a maximum peak at 18 months. On this occasion, the interspecific hybrids were more distributed between the Rs1 and Rs2 groups, and not as clustered as in the case of rubber, which was especially classified in G1.

Thus, Rs1 accessions would produce resin both in summer and winter, Rs2 members would produce resin in summer, and Rs3 members would accumulate resin in summer, but see a decrease during winter. These findings are in accord with a previous study by Jara et al. [2], who analyzed the pattern of production of resin throughout the vegetative cycle from 12 to 24 months and reported that winter would be the optimum harvesting time for the pure accessions AZ-1 and AZ-6, while spring would be best for hybrids and some other pure accessions. The best resin producer germplasm was the pure traditional accession 11600 in S3 (1196.3 kg ha^−1^), which belonged to G2 (Table 2). 

### 2.5. Concentration of Rubber and Resin 

**Figure 4:**
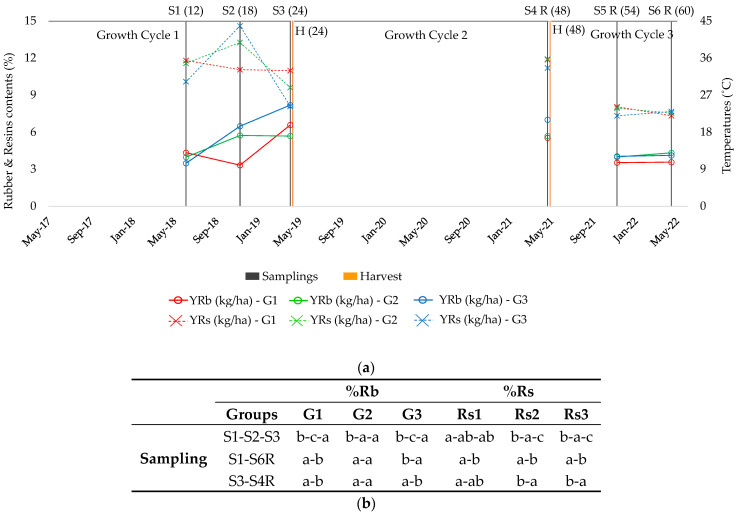
Percentage of rubber and resin (%Rb and %Rs) according to the groups (G1, G2 and G3 for Rb; Rs1, Rs2 and Rs3 for Rs) established in relation to the sampling times (S1–S3) within the first growth cycle, prior to harvesting. (**a**) Graphic representation of the three cycles; (**b**) Significant differences found with ANOVA within samplings in the first cycle of growth (S1–S2–S3) and comparing plants of the same age before and after harvesting (S1 versus S6R and S3 versus S4R). S1 (12), May 2018 (12-month-old plants); S2 (18), November 2018 (18-month-old plants); S3 (24), April 2019 (24-month-old plants); S4R (48), April 2021 (48 months after transplanting, 24 months after harvesting); S5R (54), December 2021 (54 months after transplanting, 6 months after harvesting); S6R (60), April 2022 (60 months after transplanting, 12 months after harvesting). Different letters indicate significant differences among samplings. Tukey test, *p* < 0.05.

Established models typically refer to the yield per hectare, which is a relevant parameter for both the producer and for assessing the crop feasibility. However, knowing how the content of the co-products changes might be crucial for a better understanding of plant behavior (Figure 4). Rubber content (%) evolution was similar to rubber production in the three classified groups (Figure 2): G1 accumulated rubber in winter, G2 in summer and G3 in both summer and winter. Plants of the same age behaved differently along the cycles, especially 12-month-old plants (S1 and S6R), as those belonging to G1 showed a decrease in the rubber content after the first harvest, whereas the rubber content was unchanged in G2 plants, and increased in G3 plants. In the case of 24-month-old plants (S3 and S4R), the rubber content in G2 did not significantly vary, whereas G1 and G3 plants showed a decrease in content. Notably, the rubber percentage decreased as the plant grew, which might be due to the dilution effect of the biomass, which increased one year after harvesting [2]. This also occurred in G1 plants at both 12 and 24 months old, and in G3 plants only at 24 months old.

In contrast to rubber, the evolution of the resin content was different to that established for resin production in the classified groups (Figure 3). The content in Rs1 (%, Figure 4) remained constant while the yield always increased (Figure 3). The resin content in Rs2 (%) increased considerably in summer and decreased significantly during winter, but the total yield remained constant due to the greater biomass production (Figure 3). Finally, the resin content in Rs3 was at its maximum in S2, but the lowest percentage occurred in S3 (Figure 4) instead of S1 when the yield was considered (Figure 3); this is logical considering that the biomass increased over time. Concerning the following two cycles, the resin percentage decreased in 12-month-old plants in the three groups, likely because the accumulation of resin starts at a precise moment after replantation or regrowth [20]. This can be corroborated when analyzing 24-month old plants, whose resin content increased in G2 and G3 and remained constant in G1. This demonstrates again that rubber and resin accumulation are not related and are dependent on the accession [3,22] and the plant growth. 

Biomass production, whose patterns (GP1, GP2, GP3 and GP4) were established for the same experimental field [2], also depended on the accession and could be related to the rubber and resin patterns. For instance, accessions in G2 for rubber (AZ-1, AZ-6, AZ-2 and R1040) that did not accumulate rubber in winter (considering yield or percentage) belonged to GP3 in the study by Jara et al. [2], and the biomass also remained constant from S2 to S3. However, in the case of N565, which did not accumulate rubber in summer (G1) and whose biomass increased in this season (GP2), a drop in rubber content was observed in S2, although this recovered in winter as the biomass remained constant while the rubber accumulated. Another example is 11591 (CL1) in G3, which accumulated rubber both in summer and winter; regarding biomass, in GP2, it increased in summer and remained constant in winter, with a drop in the percentage but not in the yield observed in S2, likely due to a quicker increase in biomass in summer in comparison with the accumulation of rubber. In winter, the biomass remained constant while the rubber accumulated, consequently increasing both the percentage and the yield. This relation with biomass was also observed for resin. For example, the accessions CAL-1, A48118 and 593, which belong to Rs1, maintained a stable resin content along the cycle but showed a gradual increase in yield, which is in accord with their GP4 biomass pattern, with a gradual increase from S1 to S3. 

**Figure 5:**
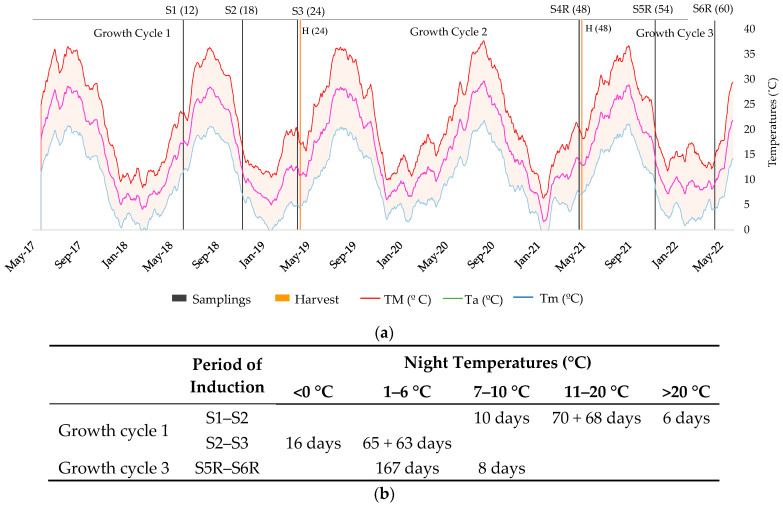
Maximum (TM), average (Ta) and minimum (Tm) temperatures: (**a**) along the three growth cycles studied; (**b**) accumulated days with different ranges of night temperatures. S1 (12), May 2018 (12-month-old plants); S2 (18), November 2018 (18-month-old plants); S3 (24), April 2019 (24-month-old plants); S4R (48), April 2021 (48 months after transplanting, 24 months after harvesting); S5R (54), December 2021 (54 months after transplanting, 6 months after harvesting); S6R (60), April 2022 (60 months after transplanting, 12 months after harvesting).

Similar to what was observed for biomass in the study by Jara et al. [2], rubber production was unaffected by Filomena, which damaged some guayule accessions [32], and actually increased in most accessions (S4R versus S3 and S6R versus S1) (Table 3). In the third growth cycle, after the second harvest, all the groups maintained their rubber and resin content from S5R (December) to S6R (April), a period in which the weather was cold, although less so than in previous growth cycles. However, the resin content in Rs2 and Rs3 was higher after harvesting in 24-month-old plants (S4R versus S3) and was lower in all three groups in 12-month-old plants (S6R versus S1). The main difference in this third growth cycle in comparison with the first and second cycles is that the minimum temperature was always above 0 °C, although cold nighttime temperatures persisted below 7 °C from S5R to S6R (Figure 5). Nonetheless, the inductive process is thought not to be continuous while low temperatures prevail [20], and may instead be activated temporarily during the winter.

The results suggest that the induction temperatures for rubber and resin production depend on the accession studied. The average cold night temperatures for the period between samples S1 and S2, which included summer, were always equal to or higher than 7 °C, with most night temperatures ranging from 11 °C to 20 °C (Figure 5b). Many accessions belonging to groups G2 and G3 could produce relevant amounts of rubber with these weather conditions, including accessions 593 and 11591 (CL1), which have been reported to be environmentally induced when night temperatures reach 20 °C over 21 days [20]. However, the induction of accessions in G1 occurred only after colder temperatures were reached, with most night temperatures ranging between 1 and 6 °C. The accessions in G3 were also induced with temperatures below 7 °C, lower than the 7–10 °C previously reported [14,15,17,19]. These hypothetical differences could be due to different factors. Chief among them is the accession, as the induction conditions have been generally tested with a small number of accessions and with younger plants. Moreover, the conditions of the study could be a factor, as they are usually controlled. Considering these results and the existing literature, the accessions in G2 would be induced with higher temperatures (11–20 °C), whereas the accessions in G1 would need temperatures below 7 °C. The accessions in G3 would be induced by both higher and lower temperatures. 

Although rubber induction has been studied extensively, there is only scarce information available on the induction of resin production. Considering the patterns established in this study (Rs1, Rs2 and Rs3), it is possible that resin induction occurs in summer for all the accessions, so would be induced with night temperatures of 11–20 °C. Only accessions belonging to Rs1 were induced in winter when the night temperatures were below 7 °C. Further research is needed to determine this temperature with precision.

The results show that the pattern observed for the different accessions in resin or rubber production is not related to the origin of the accessions (traditional or modern) and whether they are pure or hybrid.

## 3. Materials and Methods

### 3.1. Germplasm, Sampling and Monitoring

The same 27 guayule accessions and hybrids used are shown in Table 5. 

The study was established at a single location (39°53′47.26″ N; −3°07′51.87″ W) in a 0.5 ha experimental field of Santa Cruz de la Zarza. The design of the plot and the experimental characteristics of the investigation have been described previously [2], including the irrigation and fertilization regimes. The crop was studied for three cycles. The first cycle was 24 months and three sampling periods were considered (May and November 2018, and April 2019), at 13, 18 and 24 months after plantation (henceforth referred to as S1, S2 and S3). A second 24-month cycle ended in April 2021 (S4R), 48 months from transplantation. Finally, a 12-month cycle was tested, for which two samples were taken in December 2021 and April 2022 at 56 and 60 months after transplantation (S5R and S6R, respectively). 

The mean temperature trend was monitored (Figure 5a) and was calculated as the 7-day moving average of daily temperature. Santa Cruz de la Zarza is a semi-arid temperate area, with cold and dry winters, hot and dry summers, and a mild and semi-humid spring and autumn. The mean annual temperature during the study period was 15.9 °C, with a mean annual minimum temperature of 9.8 °C and with absolute minimums of up to −18.5 °C in January 2021 during the “Filomena” polar squall, which covered the crop with a layer of snow (about 50 cm thick) for two weeks. The average maximum temperature was 22.0 °C, with an absolute maximum of 42.5 °C reached several times in the hottest days of July and August across all years of the study. Overall, the average temperature was 10% above the 30-year average during the study period, with the spring of 2020 and the autumns of 2018 and 2021 being up to 12% warmer than normal. 

### 3.2. Sampling and Processing

Four representative and adjacent plants for each replication were randomly selected for each sampling. The plants were then manually harvested by cutting the stems at 10 cm above the soil and then packaged into kraft bags. Once transported to the laboratory, the samples were dried in an air oven (Selecta DryBig, Barcelona, Spain) for 48 h at 60 °C to achieve constant weight; the leaves and flowers were then manually removed. The moisture content was determined with a halogen lamp moisture analyzer (model XM-120 T; Cobos, Barcelona, Spain) at 105 °C, reaching a 11.5–12% value. The samples were ground in a centrifugal mill to a granulometry of 0.5 mm and packed into hermetic vials.

### 3.3. Resin and Natural Rubber Determination

The sequential extraction of resins and rubber was performed in a BUCHI E-914 Speed Extractor (Postfach, Switzerland). A sample of 1 ± 0.005 g of guayule was weighed and homogenized with approximately 20 g of sand as a dispersing agent to fill the stainless steel 40 mL extraction cell, leaving 1 cm of free space. A cellulose acetate filter was placed at the top and bottom of each cell to avoid sample contamination. The extraction conditions have been described in detail previously [33]. The resins and rubber extracts were collected in 250 mL flasks and transferred to pre-weighed flasks. Solvent evaporation was carried out in a Multivapor BUCHI P-6 parallel system (Barcelona, Spain) at 45 °C and 500 mbar. After evaporation, the pre-weighed flasks were maintained for 30 min in a desiccator before final weighing. The percentage of resin and natural rubber (Rs and Rb, respectively) was then determined gravimetrically. Each sample was extracted three times. The yield per hectare was used as a metric for the branch material, which was calculated for both resin (YRs) and rubber (YRb) considering a plantation density of 33,333 plants hectare^−1^. 

### 3.4. Data Analysis

The characterization of the rubber and resin content was performed in the germplasm collection for each sampling using IBM SPSS Statistics v25 [34], considering germplasm as a random effect and using the Least Significant Difference (LSD) to assess the magnitude of statistically detectable differences. Analysis of variance (ANOVA) was used to compare the evolution of production in branches (YRb and YRs) along the harvesting times, establishing groups based on the statistical significance of rubber and resin accumulation in the first growth cycle (S1–S3) using the Tukey test. ANOVA following a Tukey test was also used to compare the rubber and resin production (YRb and YRs) of plants of the same age before and after harvesting. Finally, canonical discriminant functions were used to validate the resin groups.

## 4. Conclusions

Three patterns of rubber accumulation have been established in the present study. G1 (CFS17-2005, 11600, 11604, 11619, 11635, 11693, 11701, A48118, AZ-5, R1108, CAL-7, CAL-1, CAL-2, AZ-3, R1100, R1101 and R1103) accumulates rubber in winter, G2 (AZ-1, AZ-6, R1092, R1093, R1040 and AZ-2) accumulates rubber in summer, and G3 (11591 (CL1), CFS18-2005 and 593) accumulates rubber in both summer and winter. As the production of resin is different in accessions belonging to the same groups based on rubber production, a further three independent groups were established to classify the resin accumulation profiles: Rs1 accessions produce resin in summer and winter, Rs2 accessions produce resin in summer and Rs3 accessions accumulate resin during the summer. The temperatures reached during the winter (below 7 °C) could induce rubber production in accessions in G1 and G3, while temperatures between 11 and 20 °C would be sufficient for accessions belonging to G2 and also G3. Concerning resin production, it appeared to be induced with night temperatures between 11 and 20 °C for all accessions, although those in Rs1 could also be induced with lower temperatures (below 7 °C). 

The yields of rubber and resin in the geographical area of southeastern Spain are lower than those obtained in previous studies due to the agroclimatic conditions. The improvements that would be obtained by applying a greater amount of irrigation should be studied. Biannual harvesting could accelerate rubber accumulation in summer, although more research is needed to adapt to the current production techniques in this area. Perhaps pluriannual accession crops could achieve interesting production levels along the entire the year.

## Figures and Tables

**Table 1:** Variability in germplasm rubber and resin depending on the sampling period (S1–S6R).

			Rb (%)	YRb (kg ha^−1^)	Rs (%)	YRs (kg ha^−1^)
New plantation						
Month 13	S1	mean	4.17	60.16	11.55	167.48
		min	1.84	14.25	6.81	45.64
		max	8.52	154.82	16.25	398.27
		LSD	0.35	4.41	0.54	10.56
Month 18	S2	mean	4.21	134.60	12.54	421.85
		min	1.76	3.11	6.89	11.08
		max	8.11	500.73	19.36	1707.50
		LSD	0.40	13.68	0.66	40.35
Month 24	S3	mean	6.56	303.59	10.01	479.12
		min	1.71	2.43	5.52	6.86
		max	11.60	1251.14	17.14	2415.19
		LSD	0.54	36.06	0.52	58.41
First harvest Month 25						
Regrowth Month 48	S4R	mean	5.74	341.02	11.84	720.21
		min	2.54	13.75	7.18	39.44
		max	9.10	1315.35	19.93	5079.13
		LSD	0.41	31.14	0.58	73.04
Second harvest Month 49						
Regrowth Month 56	S5R	mean	4.70	233.85	10.38	504.00
		min	2.00	6.97	6.96	13.54
		max	6.94	1974.77	13.21	3015.00
		LSD	0.31	31.87	0.35	56.11
Month 60	S6R	mean	3.80	174.42	7.48	348.93
		min	0.87	5.19	3.85	17.57
		max	6.36	757.21	10.24	1694.04
		LSD	0.27	18.24	0.34	40.26

S1, May 2018; S2, November 2018; S3, April 2019; S4R, April 2021; S5R, December 2021; S6R, April 2022; Rb, % Rubber; YRb, Branches Yield Rubber; Rs, % Resin; YRs, Branches Yield Resin.

**Table 2:** Rubber production of 27 guayule accessions across the sampling period (S1-S6R).

Accessions	Rb (%)						YRb (kg ha^−1^)					
	S1	S2	S3	S4R	S5R	S6R	S1	S2	S3	S4R	S5R	S6R
CFS17-2005	4.14	4.69	8.81	6.87	3.17	5.41	49.17	84.90	463.60	310.81	64.60	148.18
CFS18-2005	3.54	6.15	6.73	6.53	3.84	3.10	52.54	139.44	265.50	345.52	136.10	157.05
593	3.76	6.49	7.56	7.27	4.17	4.88	51.64	191.65	329.04	393.68	190.18	172.16
11590 (CL1)	3.15	6.82	10.28	7.21	4.07	4.42	40.55	211.54	428.81	324.02	221.96	194.86
11600	4.51	2.20	5.98	7.97	3.56	3.90	88.97	81.91	423.48	560.70	192.33	234.28
11604	3.86	3.57	6.40	8.72	4.04	4.44	40.52	116.16	310.77	412.53	284.03	273.35
11619	4.35	5.42	7.95	6.85	4.11	3.67	54.52	159.59	312.05	476.69	116.96	173.91
11635	5.39	3.95	11.39	6.65	2.81	2.36	93.97	121.80	566.40	316.13	169.25	134.77
11693	7.91	2.16	5.62	7.63	4.27	2.67	107.97	87.74	294.41	480.59	107.05	117.37
11701	2.51	3.18	3.44	5.55	2.51	2.47	31.39	99.05	184.38	463.21	148.50	117.52
N565	6.15	6.44	10.08	7.26	4.43	3.96	82.06	160.81	400.09	421.66	154.92	140.77
A48118	6.20	3.28	10.26	5.84	4.98	6.23	76.50	88.12	602.09	314.62	162.54	226.70
CAL-1	2.48	3.60	3.31	2.80	3.62	3.02	40.58	142.66	249.72	118.94	141.14	151.22
CAL-2	4.14	2.17	3.30	3.03	3.97	0.92	83.24	87.36	180.70	192.28	286.13	55.74
CAL-7	2.48	1.90	5.51	3.81	2.21	2.73	46.03	57.44	234.03	363.36	214.11	163.72
AZ-1	7.37	6.14	6.05	6.34	4.19	6.02	91.80	281.22	261.83	322.44	198.77	240.25
AZ-2	2.97	3.81	3.97	5.15	6.39	4.30	47.55	175.49	142.45	421.38	368.77	364.00
AZ-3	3.98	1.91	6.48	3.96	1.54	2.97	82.36	93.43	359.86	258.31	97.24	149.14
AZ-5	4.27	4.62	9.02	7.36	3.61	5.31	63.48	145.39	517.38	413.81	241.88	276.36
AZ-6	4.31	7.42	8.05	7.51	2.61	3.42	67.94	237.15	276.29	327.24	96.56	150.41
R1040	4.39	7.22	8.05	6.46	5.08	4.81	77.97	387.36	484.22	326.43	413.44	295.26
R1092	2.99	4.62	3.03	4.36	3.86	3.41	24.89	74.50	106.33	249.44	102.29	122.36
R1093	1.87	5.16	4.94	4.32	3.21	3.89	19.65	106.45	172.71	171.68	123.87	115.54
R1100	3.29	2.85	6.53	3.98	2.95	3.28	49.08	83.65	186.02	188.58	132.54	119.33
R1101	1.96	1.78	5.00	2.64	2.70	3.57	28.12	53.94	193.68	136.87	150.90	141.30
R1103	3.95	1.96	1.84	3.20	5.88	2.41	51.83	61.00	94.54	162.35	254.71	119.07
R1108	6.61	4.26	6.44	5.60	3.96	5.26	72.73	84.45	178.92	218.57	116.50	166.06
mean	4.17	4.21	6.56	5.74	4.70	3.80	60.16	134.60	303.59	341.02	233.85	174.42
LSD	0.35	0.40	0.54	0.41	0.32	0.31	6.15	20.10	52.74	37.13	41.56	22.81
Sig.	***	***	***	***	***	***	***	***	***	***	*	***

S1, May 2018; S2, November 2018; S3, April 2019; S4R, April 2021; S5R, December 2021; S6R, April 2022; Rb, % Rubber; YRb, Branches Yield Rubber. LSD: mean deviation or minimal significant difference; * *p* < 0.05, *** *p* < 0.001.

**Table 3:** Resin production of 27 guayule accessions across the sampling period (S1–S6R).

Accessions	Rs (%)						YRs (kg ha^−1^)					
	S1	S2	S3	S4R	S5R	S6R	S1	S2	S3	S4R	S5R	S6R
CFS17-2005	9.78	10.08	7.00	9.41	7.67	6.95	116.21	182.63	368.34	425.91	148.17	190.48
CFS18-2005	10.30	8.71	8.30	9.77	7.53	6.78	152.96	197.53	327.14	516.40	255.99	346.29
593	6.89	6.89	5.57	7.21	4.76	5.07	94.54	203.27	242.19	390.12	216.41	178.73
11590 (CL1)	11.23	10.28	9.86	9.01	7.16	6.57	144.42	319.03	411.27	404.85	368.09	294.68
11600	11.14	14.82	16.90	12.78	10.77	5.39	220.00	552.83	1196.34	899.19	586.21	327.06
11604	11.66	16.00	9.07	12.50	8.58	7.41	122.43	520.76	440.34	591.23	558.65	452.57
11619	14.24	14.88	11.65	11.80	8.69	8.49	178.49	438.49	457.25	820.79	257.94	408.80
11635	11.08	14.69	11.41	13.74	8.00	7.31	192.94	452.78	567.22	652.73	501.17	413.97
11693	15.35	13.77	10.32	13.06	9.88	7.23	209.54	559.27	540.59	822.49	249.12	317.10
11701	14.10	11.33	13.01	13.03	7.35	7.86	176.44	352.45	697.81	1078.39	409.86	362.04
N565	7.11	13.07	10.99	10.04	10.34	6.10	94.89	326.18	436.04	583.64	336.54	216.95
A48118	14.55	13.95	12.24	11.99	8.17	9.34	179.65	374.93	718.19	645.57	260.68	340.25
CAL-1	10.25	11.79	7.75	8.81	6.90	6.09	167.83	466.78	553.60	374.96	260.33	305.10
CAL-2	11.43	7.70	8.43	9.57	8.46	3.99	229.93	309.57	465.13	607.05	645.85	241.56
CAL-7	12.43	12.87	10.05	16.35	9.07	8.81	230.27	388.99	426.90	1559.26	745.03	528.18
AZ-1	11.48	16.69	8.67	12.87	8.01	9.45	143.03	765.00	375.00	654.47	367.10	376.99
AZ-2	9.73	12.92	10.87	14.36	8.67	9.80	155.76	598.42	389.94	1174.73	528.04	813.00
AZ-3	14.17	13.00	11.23	15.31	9.89	8.85	292.97	636.78	623.30	997.42	608.58	446.86
AZ-5	14.57	19.25	10.92	16.23	7.83	9.06	216.71	606.04	626.53	912.53	499.39	471.52
AZ-6	8.70	12.50	7.52	9.51	6.67	5.88	137.17	399.66	258.11	414.25	238.13	258.49
R1040	12.57	13.91	9.25	13.37	8.74	8.62	223.51	751.69	556.70	675.40	730.59	528.34
R1092	9.57	9.46	12.79	12.49	7.88	8.36	79.75	152.52	449.14	715.33	224.47	299.50
R1093	12.04	10.32	10.77	15.61	7.15	10.08	126.78	209.28	376.08	620.71	303.85	299.21
R1100	13.17	10.36	8.15	9.11	5.37	5.71	196.58	304.34	231.95	431.88	263.35	207.95
R1101	7.69	11.36	10.56	10.34	10.01	8.66	110.44	344.12	409.15	536.99	571.05	342.25
R1103	11.42	9.84	6.55	10.86	7.08	6.90	149.70	305.69	336.73	551.28	306.04	340.17
R1108	15.18	18.11	10.53	10.67	9.07	7.57	167.05	359.17	292.55	416.13	248.08	239.38
mean	11.55	12.54	10.01	11.84	10.38	7.48	167.48	421.85	479.12	720.21	504.00	348.93
LSD	0.54	0.68	0.52	0.60	0.44	0.38	15.08	55.86	77.93	82.03	82.64	42.85
Sig.	***	***	***	***	***	***	***	***	**	***	NS	***

S1, May 2018; S2, November 2018; S3, April 2019; S4R, April 2021; S5R, December 2021; S6R, April 2022; Rs, % Resin; YRs, Branches Yield Resin. LSD: mean deviation or minimal significant difference; ** *p* < 0.01, *** *p* < 0.001, NS: no significant difference.

**Table 4:** Classification of accessions based on the accumulation of rubber production in the first growth cycle and comparison between plants of the same age before and after harvesting.

Groups	Accessions	Sampling						
		Growth Cycle 1			24-Month-Old Plants		12-Month-Old Plants	
		S1	S2	S3	S3	S4R	S1	S6R
G1	CFS17-2005	c	bc	a	a	a	b	a
	11600	b	b	a	b	a	b	a
	11604	c	bc	ab	b	a	b	a
	11619	c	bc	ab	b	a	b	a
	11635	b	b	a	a	b	a	a
	11693	c	c	b	b	a	a	a
	11701	b	b	b	b	a	ab	a
	N565	b	b	a	ab	a	ab	a
	A48118	d	d	a	a	b	b	a
	CAL-1	b	b	a	a	b	b	a
	CAL-2	cd	bcd	ab	a	a	a	ab
	AZ-3	c	c	a	a	a	b	a
	AZ-5	c	c	ab	a	a	b	a
	R1100	c	c	a	a	a	b	a
	R1101	c	c	a	a	b	b	a
	R1103	c	c	c	b	a	b	a
	R1108	b	b	a	ab	a	b	a
	CAL-7	c	bc	a	a	a	b	a
G2	AZ-6	c	ab	a	a	a	b	a
	AZ-1	c	ab	ab	b	a	b	a
	AZ-2	b	ab	ab	b	a	b	a
	R1040	b	ab	a	a	a	b	a
	R1092	b	ab	ab	b	a	b	a
	R1093	c	b	ab	b	a	b	a
G3	11591 (CL1)	d	bc	a	a	ab	b	a
	CFS18-2005	c	bc	ab	ab	a	b	a
	593	d	c	b	b	a	b	a

G1, G2, G3: groups 1, 2 and 3 based on the significant differences found with ANOVA within samplings in the first cycle of growth; S1, May 2018; S2, November 2018; S3, April 2019; S4R, April 2021; S5R, December 2021; and S6R, April 2022; S4R and S6R: Regrowth after harvesting. Different letters indicate significant differences among samplings. Tukey test, *p* value <0.05.

**Table 5:** Guayule accessions studied in Santa Cruz de la Zarza (Toledo, Spain).

Accession	Species	Seed Code	Origin	Year	Ploidy
Traditional guayule pure accessions					
A48118	*P. argentatum*	PI 478662 13i SO	Durango, México	1948	3×, 4×, 4.5×
593	*P. argentatum*	PI 478639 13i SO	IRC	1926	4×
11600	*P. argentatum*	PI 478641 15i SO	ERP, 4265-I	1950	3×, 4×
11604	*P. argentatum*	PI 478642 12i SO	ERP, 4265-I	1950	4×
11619	*P. argentatum*	PI 478645 12i SO	ERP, 4265-I	1950	3×, 3.8×, 4×
11635	*P. argentatum*	PI 478648 15i SO	ERP, 4265-I	1950	2×, 4×
11693	*P. argentatum*	PI 478650 12i SO	ERP, 4265-I	1950	3.8×, 4×
11701	*P. argentatum*	PI 478651 12i SO	ERP, 4265-I	1950	4×
N-565	*P. argentatum*	PI 478655 15i SO	ERP, 4265-I	1950	3×, 4×, 5.7×
Modern guayule pure accessions					
R-1040	*P. argentatum*	W6 2192 15i SO	Coahuila, México	1976	3.7×, 4×
R-1092	*P. argentatum*	W6 2244 12i SO	Durango, México	1979	3×, 4×
R-1093	*P. argentatum*	W6 2245 15i SO	Durango, México	1979	3×, 3.7×
R-1108	*P. argentatum*	W6 2260 13i SO	Texas, USA	1981	3×, 4×
CAL-7	*P. argentatum*	W6 715715i SO	USDA-CA	1985	4×
AZ-1	*P. argentatum*	PI 599674 13i SO	USDA-AZ	1997	4×
AZ-5	*P. argentatum*	PI 599678 13i SO	USDA-AZ	1997	4×, 4.4×
CFS17-2005	*P. argentatum*	PARL 804 15i SO	Texas, USA	2005	4×
CFS18-2005	*P. argentatum*	PARL 805 15i SO	Texas, USA	2005	3× (3), 4× (3)
11591 (CL-1)	*P. argentatum*	CL1	CIRAD	2017	3×
AZ-6	*P. argentatum*	CL6	CIRAD	2017	4×
Modern hybrid accessions					
R-1103	*P. argentatum* × *P. incanum*	W6 2255 13i SO	Durango, México	1977	4×
R-1100	*P. argentatum* × *P. incanum*	W6 2252 13i SO	Coahuila, México	1979	4×
R-1101	*P. argentatum* × *P. incanum*	W6 2253 15i SO	Coahuila, México	1979	4×
CAL-1	*P. argentatum* × *P. tomentosum*	PI 478666 13i SO	USDA-CA	1982	3×, 4.5×, 7×
CAL-2	*P. argentatum* × *fructicosum*	PI 478667 13i S	USDA-CA	1982	3×, 4×
AZ-2	*P. argentatum* × *unknown*	PI 599675 13i SO	USDA-AZ	1997	4×
AZ-3	*P. argentatum* × *unknown*	PI 599676 12i SO	USDA-AZ	1997	4×, 6×

## Data Availability

Data are contained within the article.
